# What's in It for Me? Contextualizing the Potential Clinical Impacts of Lecanemab, Donanemab, and Other Anti-β-amyloid Monoclonal Antibodies in Early Alzheimer's Disease

**DOI:** 10.1523/ENEURO.0088-24.2024

**Published:** 2024-09-26

**Authors:** Michelle Jin, James M. Noble

**Affiliations:** ^1^Medical Scientist Training Program (MSTP), Columbia University Irving Medical Center (CUIMC), New York, New York 10032; ^2^Neurobiology and Behavior (NB&B) Graduate Program, Columbia University, New York, New York 10027; ^3^Department of Neurology, Taub Institute for Research on Alzheimer’s Disease and the Aging Brain, and the GH Sergievsky Center, Columbia University Irving Medical Center, New York, New York 10032

**Keywords:** Alzheimer’s disease, clinical efficacy, donanemab, lecanemab, minimal clinically important difference

## Abstract

A new era of disease-modifying therapy for Alzheimer's disease (AD) arrived in 2021 following the Food and Drug Administration's (FDA) decision to grant accelerated approval for aducanumab, an anti-β-amyloid (Aβ) monoclonal antibody designed to target Aβ aggregates, a biological component of AD. More recently, trial outcomes for lecanemab and donanemab, two additional antibodies of this drug class, have shown favorable and significant slowing of metrics for cognitive and functional decline. Lecanemab and donanemab have since received similar FDA approval to aducanumab in January 2023 and July 2024, respectively. Given that these therapies are a clearly emerging tool in the repertoire of clinicians treating AD and related dementias, a critical dialogue has been ongoing regarding the potential impacts and place for these therapies. Here, we seek to contextualize this debate by first considering factors involved in theoretically extrapolating current randomized control trial outcomes to estimate meaningful clinical impacts. In the process of this exercise, we outline a generally useful concept termed Summative Treatment-Associated Benefit measuring Long-term Efficacy/Effectiveness Area as a metric of summative benefits of treatment over the life course of an individual. Second, we consider current real-world factors, such as conditions of FDA approval and other points involved in clinical decision-making that will influence and/or temper the actual impacts of this drug class.

## Significance Statement

Newly available disease-modifying therapies for Alzheimer's disease which target β-amyloid, a biological component of the disease, are quickly becoming a part of the therapeutic repertoire of clinicians treating patients with dementias. While recent outcomes of clinical trials evaluating this treatment class show a favorable slowing of cognitive decline, there remains an ongoing dialogue regarding the potential of these therapies to lead to meaningful clinical changes long term. Here, we provide perspective on this topic by highlighting the major factors involved in theoretically extrapolating recent trial outcomes in the context of disease staging and disease trajectory. We then point to considerations, including the safety profile, burden of administration, and Food and Drug Administration approval, that will influence the real-world impacts of this drug class.

## Introduction

In June 2021, disease-modifying treatments (DMTs) for Alzheimer's disease (AD) first became available following the United States Food and Drug Administration's (FDA) approval for aducanumab, one of the several passive immunotherapy anti-β-amyloid (Aβ) monoclonal antibodies (MABs; Cavazzoni, 2021) designed to relieve the deposition of Aβ aggregates, a recognized biological component of AD. Approval of aducanumab followed completion of two similar Phase 3 double-blind randomized controlled trials (RCTs). While both trials were initially terminated following a futility analysis concluding no apparent slowing of cognitive or functional decline (Knopman et al., 2021), a subsequent long-term analysis suggested a favorable clinical outcome in one trial but not the other (Cummings et al., 2021). This was perhaps due to a trial design difference involving dose escalations toward the end of the enrollment period. These results, along with a broader acceptance of AD biomarkers as surrogate measures of clinical improvement (in which a “reduction in plaques is reasonably likely to result in clinical benefit”; Cavazzoni, 2021), were central to advisory panel deliberations and ultimately conditional approval requiring continued study in a new RCT. Collectively, results of the aducanumab trials reflected outcomes of several other anti-Aβ MAB trials demonstrating variable levels of convincing amyloid target engagement but disappointing clinical impacts (Salloway et al., 2014, 2021; Honig et al., 2018; Bateman et al., 2023).

More recently, two more RCTs for anti-Aβ MABs were completed, with the outcomes of the CLARITY-AD trial for lecanemab published in November 2022 (van Dyck et al., 2023) and the TRAILBLAZER-ALZ 2 trial for donanemab published in August 2023 (Sims et al., 2023). Overall, results favored a clinical benefit, as both trials met primary and secondary endpoints for slowing cognitive and functional decline, along with now-expected decreases in AD biomarkers. In January 2023 and July 2024, respectively, lecanemab and donanemab received similar FDA approval to aducanumab. Despite these developments, aside from barriers to treatment and prescription access, anti-Aβ MABs continue to face challenges with adoption and comfort by some clinicians and patients. It is recognized that the effects on slowing cognitive decline are modest (Ackley et al., 2021) and the durability of effect is uncertain at this time until long-term outcomes phases of study are complete. Additionally, each anti-Aβ MAB has recognized complications of amyloid-related imaging abnormalities (ARIA), which have occurred to varying degrees of frequency and severity and most often within the first year of treatment. With the current real-world rollout of lecanemab and donanemab and follow-up of treatment outcomes with registry-based data collection, critical questions regarding the long-term effectiveness of anti-Aβ MABs may soon be addressed.

The objectives of this commentary are to: (1) better contextualize the cognitive outcomes of these trials in a relatable framework to the lay public, (2) highlight important considerations involved in their extrapolation long-term, taking factors such as disease staging and time benefits into account when considering the critical question of whether these DMTs will make a meaningful clinical difference in AD patients, and (3) summarize the major clinical and logistical factors that must be considered under this current phase of real-world widescale implementation of anti-Aβ MAB therapies that will influence actual impacts of this drug class. While framed around the results of completed anti-Aβ MAB studies, the factors under consideration may be applicable to other forthcoming therapies in AD and hopefully therapies still on the horizon for other neurodegenerative diseases.

### Summarization and interpretation of trial outcomes

As a brief overview of the similarities and differences in trial designs, both the lecanemab and donanemab trials followed participants for ∼18 months, and both studies enrolled participants with mild cognitive impairment (MCI) or mild dementia due to AD. Persons with early-stage disease were selected for treatment because of evidence suggesting earlier intervention with DMTs is most promising in producing a biological impact reflected through clinical indicators. Moreover, there is hardly any human illness in which time to treatment does not matter and where later phase treatments demonstrate better outcomes than early-stage treatments. This notion is independent of the point that an effective clinical regimen requires stage-specific biological and clinical target engagement.

Trial differences included the frequency of dosing—lecanemab infusions are biweekly infusions whereas donanemab are monthly—and the primary cognitive scales used. CLARITY-AD employed the Clinical Dementia Rating (CDR)–Sum of Boxes (CDR-SB), with scores ranging from 0 to 18 (higher score being worse), whereas TRAILBLAZER-ALZ 2 used the integrated AD rating scale (iADRS), with scores ranging from 144 to 0 (lower scores being worse; Wessels et al., 2015, 2022). Both are generally well-validated measures that reflect both cognitive performance and impact on daily function.

Differences in design of these two anti-Aβ MABs RCTs offer more nuanced insight on critical questions regarding duration and schedule of treatment: whether anti-Aβ MAB should be prescribed for a fixed amount of time at a fixed dose, until a targeted threshold is reached, or whether treatment should follow an induction and maintenance model. The donanemab trial included a “treat until clearance” strategy in which donanemab arm participants were switched to placebo once they met a predefined threshold of Aβ clearance. In contrast, lecanemab study participants were treated on a prespecified fixed timeline, independent of incident biomarkers. The donanemab trial also offered insights into treatment efficacy relative to stage of illness by stratifying groups into low/medium versus high tau groups based on tau-PET imaging.

Outcomes of the CLARITY-AD trial showed the lecanemab arm had an adjusted mean score change from the baseline of 1.21 and the placebo arm had a change of 1.66, resulting in a relative worsening of 0.45 points on the CDR-SB. In TRAILBLAZER-ALZ 2, the donanemab low/medium tau arm had an adjusted mean decline of −6.02 from the baseline, whereas the placebo arm declined by −9.27, resulting in a relative worsening of −3.25 points on the iADRS. While these effects appear modest, it is important to frame their magnitude in the context of the disease course. The cognitive score trajectories in persons treated with anti-Aβ MABs reflect two phases: disease impacts before and after treatment. Measuring a drug effect leading to slowing of cognitive decline can only be appropriately made in relation to the baseline cognitive state at the start of treatment. The results of lecanemab and donanemab convincingly show slowing of decline, with lecanemab resulting in 27% less cognitive decline and roughly a 6-month delay in disease progression (van Dyck et al., 2023) and donanemab resulting in a 35% decrease in cognitive decline and a 4.36-month delay in disease progression (Sims et al., 2023). An 18-month study period is brief within the broader consideration of the, likely decades-long, AD time course. Additionally, recognizing the realities of practical trial lengths and the importance of establishing a starting point for the field, a 6-month delay achieved over this study period is not insignificant.

An aspirational effect of these or any AD therapy is the complete arrest of cognitive decline or even recovery. No anti-Aβ MABs have demonstrated either outcome, although some trials including TRAILBLAZER-ALZ 2 included analyses on the proportion of treated participants who had no decline (Sims et al., 2023). In many conditions, neurological or, otherwise, full recovery is not only unrealistic but not expected by patients whose goals are often centered on remaining the same as they are now. Furthermore, it is established that AD neuropathology begins years to decades prior to first symptoms, which are likely further buffered by network-level compensatory mechanisms. Therefore, current strategies of using therapies only at the symptomatic stage are unlikely to reverse years or even decades of accumulated neuropathological insult. Hence, the best and most realistic possible outcomes of current approaches may be to slow cognitive decline. Nonetheless, this is consistent with other stage-dependent treatment models in other chronic illness and provides an apparent advantage over previously available treatments for early stage of AD. In particular, acetylcholinesterase inhibitors provide a modest and brief symptomatic benefit but ultimately are not thought to slow disease progression (Marucci et al., 2021).

### Interpreting minimal clinically important differences

An important question to consider is: From whose perspective should a therapeutic effect be judged—from the perspectives of patients, caregivers, and treating clinicians or the financial viewpoints of care expenditure analyses tracked through administrative claims registries? Pragmatically, all aspects have to be considered to demonstrate a feasible, durable place in healthcare. But clearly, the most critical stakeholder is the patient. For this purpose, one commonly used metric is the minimal clinically important difference (MCID). The MCID is the minimal change in score that would be perceived as altered cognition/function (although it is estimated from structured clinician assessments). The MCID been estimated for different cognitive scales (Andrews et al., 2019; Wessels et al., 2022; Lansdall et al., 2023), and it represents within-patient changes in clinical status. Notably, it is not meant to be applied as a threshold for evaluating clinical efficacy of treatment arm differences. FDA guidelines state that “a treatment effect is different from a meaningful within-patient change. The terms MCID and minimum important difference do not define meaningful within-patient change if derived from group-level data” (US Food and Drug Administration, 2019). Attempts to apply MCID to group-level analyses of AD DMT trials present risks for inflating the magnitude of treatment–placebo arm differences needed to demonstrate clinical efficacy.

To illustrate why, consider a hypothetical scenario whereby a novel candidate drug immediately halted disease progression, sustaining function at a baseline level—a remarkable outcome at early-stage disease. One can draw upon figures from the lecanemab and donanemab trials to explore this scenario further. The MCID in MCI is estimated to be one for CDR-SB (Andrews et al., 2019) and five for iADRS (Wessels et al., 2022). The time for the placebo arm in the lecanemab trial to worsen by one CDR-SB point from the baseline would take ∼10.5 months (based on Fig. 2*A* of the CLARITY-AD trial publication; van Dyck et al., 2023), and the time for the placebo arm in the donanemab trial to worsen by five iADRS points from the baseline would take ∼12 months (∼52 weeks; based on Fig. 2*A* of the TRAILBLAZER-ALZ 2 publication; Sims et al., 2023). In both cases, it would take close to 60–70% of the entire trial time just to demonstrate efficacy of a perfect disease-halting effect if MCID were applied at the group level.

Based on established pathophysiological and pharmacokinetic data, it takes time for MABs to work, and no available DMT causes immediate or rapid resolution of pathology upon initiation. The above scenario again highlights the slowly progressive nature of cognitive decline during this early disease stage relative to the short timeline of the RCTs published. Instead, the time to MCID shown either as a survival curve or cumulative distribution function or responder/progressor proportion analyses are more appropriate way to use this construct. These types of analyses help us answer questions such as: For what proportion of patients is quality of health preserved without a significant clinical change? Based on distribution data, by what time margin can most treated patients expect to preserve their current status?

### Estimating potential summative clinical benefits

**Figure 1.**
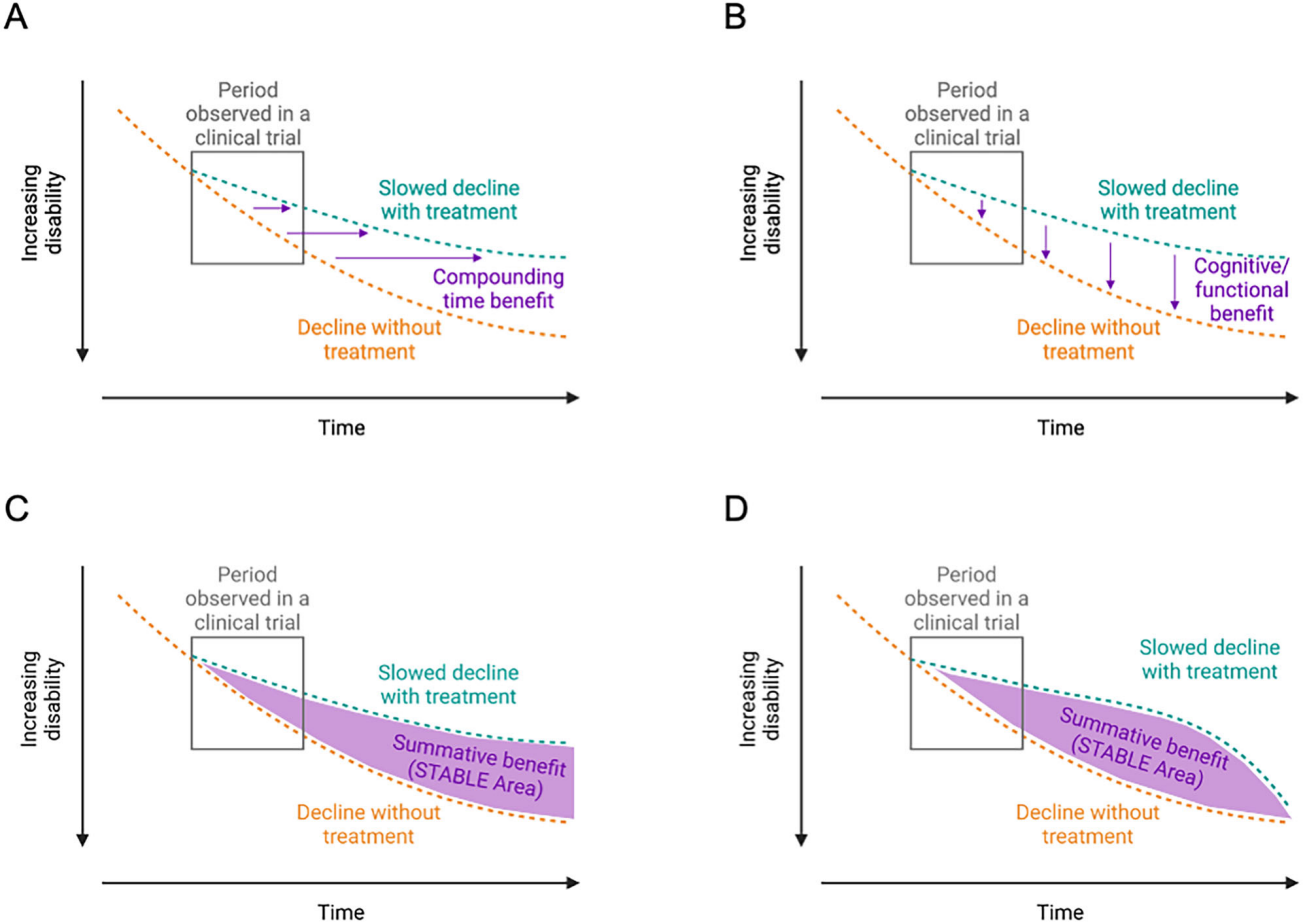
Clinical benefits associated with slowing of cognitive decline. ***A***, Assuming a consistent benefit of treatment with time, the delay to reach the same degree of cognitive impairment compounds with time in the treatment arm. ***B***, The cognitive difference between treatment and placebo arms may compound with time. (Figure adapted from Assunção et al., 2022, Fig. 2 ). ***C***, Summative clinical benefits are composed of both the time benefit and cognitive benefit. It is calculated as the area between the normal decline trajectory without treatment and the slowed decline with treatment. ***D***, Possible summative clinical benefits in the event of a time-limited clinical effect of treatment.

Another central question to treatment of any condition is: How clinically meaningful are these effects? To fully answer this, clinical benefits can be divided into two components: (1) time benefits and (2) cognitive benefits. It is recognized that an 18 month course of treatment is brief relative to the trajectory of early-stage AD (Fig. 1). Therefore, framing clinical impacts solely based on trial scale endpoint differences neglects the potentially more relatable readout of time in a slow-progressing disease. Framing clinical benefits as scale-dependent or time improvements attempts to relate the epidemiological concept of effect estimation when considering the counterfactual condition (Lash et al., 2021)—that is, the best estimate of what would have happened had the person not had their intervention, in this case not receiving anti-Aβ MAB, if all other life variables remained equal.

An integrative metric exists in the field of healthcare policy, where patient-based metrics of health-related quality of life are assessed as quality-adjusted life years (QALY), a standardized unit of health benefit whereby one unit represents 1 year of perfect health (Kaplan and Hays, 2022). Often QALYs are used to investigate benefits centered on healthcare cost-utility and savings. However, QALYs do not readily translate to individual disease outcomes important to patients and families. While helpful in informing policy, QALYs are not easily converted to personalized valuations of cognitive and psychosocial impacts of disease over time which may inform individual care decisions and clinical situations. Nonetheless, QALYs provide a helpful framework to measure clinical benefit by establishing a valuable combined time–outcome metric in healthcare policy decision-making.

To facilitate improved personalized decision-making, we propose a patient-centered metric adapted to the QALY framework. The proposed term, the Summative Treatment-Associated Benefit measuring Long-term Efficacy/Effectiveness (STABLE), representing an alternate integrated area calculation of outcome over time intended to provide a multimodal level of understanding of benefit in a treatment which slows or stabilizes disease. The concept intends to move away from unidimensional and unfortunately abstract traditional study outcomes of percentage differences in disability between two groups over time. The outcome product time × ability, framed as an amount of “stability-time,” is likely more relatable for decision-making, especially if there are two or more treatments available, as is the case now with anti-Aβ MAB therapies. The term is intended to be adaptable to concepts of “efficacy” (e.g., well-controlled prospective clinical trials) and “effectiveness” (e.g., real-world assessments and post-marketing study). Given it is graphically based, a STABLE Area can be shared as a relatable visual to patients for decision-making purposes.

The following equation would be used to calculate this metric:

$$\mathrm{STABLE}\;\mathrm{Area}=\int_{\mathrm{tstart}}^{\mathrm{tQOL}}\;[\;f(t)-g(t)]\mathrm{dt}.$$

Here, f(t) represents cognition or any other functional measure, as a function of time in the treatment scenario, whereas g(t) represents the normal trajectory of decline in the untreated scenario. The STABLE Area is the difference between these functions integrated from the start of treatment (tstart) to time to a prespecified quality-of-life outcome (tQOL). Here, tQOL should ideally be defined a priori by patients themselves and/or caregivers and is the threshold at which slowing the disease course is no longer perceived as a clinical benefit because function in the measured domain (e.g., cognition, motor, etc.) no longer contributes to meaningful quality of life. Of course, this threshold is personalized for patients and/or caregivers and can range from the loss of independence to institutionalization or to death. Individual variability makes estimating tQOL and the trajectory of f(t) a difficult task. This variability presents a challenge in estimating the average, summative therapeutic benefits at a group level. Nonetheless, as a time–ability combined unit, the STABLE Area is an improved way to measure clinically meaningful change in clinical trials and relate them to patients.

Examples of the STABLE Area concept and various models are shown in Figure 1. Assuming a consistent benefit of treatment across time, one which produces a hoped-for divergence between treatment and placebo arms, Figure 1*A* illustrates how treatment slows decline and compounds with time. Differences in cognitive performance at any given point between treated and untreated arms may also increase with time, resulting in a cumulative benefit (Assunção et al., 2022), although this effect may be modest at the end of a short clinical trial (Fig. 1*B*). However, neither time delay nor cognitive difference at any given timepoint is sufficient to capture the total clinical benefits experienced. Instead, an integrated measure of treated–untreated scale differences over time incorporates both types of benefits and is a more appropriate readout to assess total clinical impact (Fig. 1*C*).

In the field of AD, for the first time, anti-Aβ MAB presents the possibility of an enduring therapeutic benefit. However, should the treatment benefit have a limited effect with time and eventually return to the level and trajectory of the nontreated state, a fourth condition may instead occur (Fig. 1*D*). In this case, trajectory is initially meaningfully slowed before a more rapid terminal decline. The shape in Figure 1*D* also reflects response curves similar to understood benefits of cholinesterase inhibitors and memantine based on their respective trials (Rogers et al., 1998; Tariot et al., 2004; Howard et al., 2012). Even in the case of a time-limited benefit, changing the trajectory may still be beneficial so long as more time is spent at a stage with higher quality of life, traded for a shorter period of more rapid decline. More generally, no matter the exact trajectory of the treated condition, so long as a large enough STABLE Area is achieved, a treatment could be considered clinically meaningful.

Determining the merits of the STABLE Area would require more formal study on how cognition and function impact quality of life over time in the AD population but could easily be adapted to studies which have already completed. Additionally, anecdotally in clinical practice, some patients and families who perceive therapeutic benefit reflect on a series of personal events which may not have been otherwise possible without treatment. That is, the value of attending a family event this week (e.g., a grandchild's dance recital) is considered along with the value of participating in another event 6 months prior (e.g., their own 50 year wedding anniversary). The STABLE Area concept values the anniversary as well as the recital, whereas data collected at the end of a trial would only value the final event—here, the recital.

Overall, relaying to patients the potential clinical benefits of anti-Aβ MAB based on outcomes of RCTs can be challenging. As much as anti-Aβ MAB RCTs may demonstrate statistical benefit in score differences across various cognitive–functional scales, translating a slowing of cognitive decline into relatable concepts in pretreatment patient counseling by explaining its impact in either time-based or test score–based discussions can be difficult to grasp. Even so, the STABLE Area concept provides a relatable and more holistic visual of the summative benefits of treatment. As anti-Aβ MAB are the only approved DMTs that target a presumed component of AD etiology, summative clinical benefits may increase as treatment effects continue across the lifetime or as additional treatment modalities are developed.

### Safety and clinical considerations

Anti-Aβ MABs are associated with established adverse events that can be potentially life-threatening. Patients are susceptible to infusion reactions and ARIAs, in the forms of edema/effusions and microhemorrhages/hemosiderosis. The most consistent risk factor for ARIA is homozygosity for the APOE ε4 allele; ARIA risk in APOE ε4 study participants is approximately double that of noncarriers, with ε4 heterozygotes having slightly higher risk (Sims et al., 2023). Based on trial data, including open-label extension phases, along with case reports (Solopova et al., 2023), ARIA risk among APOE ε4 homozygotes treated with anti-Aβ MABs related remains a concern to the field.

Additionally, concerns have been raised regarding imaging and structural volume changes following treatment. While generally accepted by the FDA as a metric of target engagement and clearance, some debate the extent which amyloid-PET tracer signal decreases truly reflect MAB-induced amyloid removal from gray matter (Høilund-Carlsen et al., 2023). Lateral ventricle enlargement, which is strongly associated with ARIA-causing MABs and is presumed to be mechanistically linked to ARIAs, has been demonstrated for both lecanemab and donanemab (Alves et al., 2023). Lecanemab, donanemab, and similar MABs also reduce whole-brain volume, including in white matter, in the case of donanemab (Alves et al., 2023). However, whether this shrinking represents true atrophy (cell death and synapse loss) or instead is a form of pseudoatrophy has been debated (Barkhof and Knopman, 2023). Ultimately, whether these structural changes are a benign and stable by-product of treatment or reflect serious long-term side effects remains to be determined.

In extrapolating this information to the real-world decision to treat, safety and efficacy profiles remain uncertain for underrepresented groups in anti-Aβ MABs trials, ranging from persons with common or chronic medical conditions as well as race and ethnic minority groups who comprised an unacceptably small fraction of those enrolled in the studies (Manly and Deters, 2023). Historically, a strong selection bias exists for recruitment of subjects to clinical dementia trials (Franzen et al., 2022), ultimately leading to either (1) a relative underrepresentation of some groups (e.g., in TRAILBLAZER-ALZ 2) or (2) relative representation of some groups but limited group-level enrollment resulting in insufficient power to determine group-wise effect or risks (e.g., in CLARITY-AD).

All considered, it is difficult to know on an individual basis who may respond most favorably to anti-Aβ MAB treatment and who will not. Whether it is reasonable to prescribe these treatments at the individual level is dependent on a variety of factors including an individual's own AD and non-AD–related cognitive aging trajectories, which reflect a myriad of factors—genetics, life-course comorbidities, concurrent medications, and demographics and other social determinants of health—combined with treatment factors including access to expert care, and support for shared decision-making between patients, caregivers, and their clinical team. For example, the burden of undergoing once or twice monthly infusions and serial MRI monitoring may be unacceptably high by detracting from other activities which the patient may prioritize (e.g., committing otherwise active leisure time to a new recurring medical activity). In-depth considerations of these factors have been previously explored (Manly and Deters, 2023; Rabinovici and La Joie, 2023; Ramanan et al., 2023).

Ultimately, in addition to communicating the medical factors and risks outlined, communication of potential summative clinical benefits in a relatable manner is also central in decision-making among anti-Aβ MAB candidates. Proper and thorough patient education is needed to assist with informed decision-making at the time of considering anti-Aβ MAB treatment.

### The current path to widescale implementation of anti-Aβ MABs

Although aducanumab was the first-to-market of the anti-Aβ MABs, from a practical standpoint, rollout was slow because of its high price and limited coverage by the Centers for Medicare and Medicaid Services (CMS). This entailed insurance coverage only for those enrolled in clinical trials, reflecting the coverage with evidence development (CED) for this class of medications. Multiple large hospital-based organizations deferred formulary inclusion of aducanumab, functionally making it accessible through mostly private outpatient infusion centers and home-based programs. The limited availability of diagnostic testing (lumbar puncture for CSF biomarkers or amyloid-PET), as well as specialists comfortable with prescribing aducanumab, further complicated implementation of this drug.

In January 2023, lecanemab received similar FDA approval to aducanumab and a slow rollout also followed. In July 2023, broader FDA approval was given along with establishment of new CED coverage by CMS which paid for 80% of the drug cost, so long as treated patients agreed to inclusion in a registry for postapproval monitoring, with reassessments every 6 months. Importantly, in Autumn 2023, CMS further revised its policy to support coverage of a single amyloid-PET image in the context of a workup for cognitive impairment. Previously CMS amyloid-PET support was only provided as part of a CED policy if someone was registered as part of a trial (whether or not it was an anti-Aβ MAB study). The net effect was that diagnostic testing in advance of treatment with lecanemab had a financial support pathway sufficiently acceptable to make lecanemab available through a broader network of clinical programs. By early January 2024, upward of 3,000 persons in the United States were known to have been registered for lecanemab treatment, and by the end of the same month (Rising Leqembi Prescriptions, 2024), Biogen announced it would no longer be supporting development of aducanumab (which never became eligible for CMS registry and related support). Most recently, in early July 2024, FDA approval was granted for donanemab (US Food and Drug Administration, 2024), broadening treatment options available to patients within this drug class. Overall, the pathway to initial FDA approval of these MABs has been challenging and, in other ways, paradigm-shifting. MAB availability has for the first time enabled patients and caregivers to take some agency over their care in what are potentially clinically impactful but also seriously risky medications.

The perspective of this commentary is intentionally separate from speculation on the population-wide socioeconomic cost–benefit ratio regarding coverage of these treatments which have been explored by others (Lin et al., 2023; Rosenthal, 2023). The consideration of whether these treatments should be available as an option to patients is a different form considering if these treatments should receive coverage by CMS or otherwise. Exercising the CMS CED is a reasonable compromise for new interventions that may be beneficial but do not necessarily meet the descriptive qualifier as “reasonable and necessary” for traditional coverage. The utilization of a prospective national registry to track treatment outcomes (Odouard et al., 2023) will likely be critical to understanding the long-term clinical benefits of these therapies and among a broader range of individuals who were not well represented in the RCTs.

## Conclusions

After decades of development, newly available DMTs are exciting to the scientific field of dementia research as well as affected and at-risk patients and their caregivers. Here, we have outlined several key factors to consider in the evaluation of potential individual and population-level clinical impacts of this class of drug based on most recent RCT outcomes. Central questions still remain around MAB treatment duration and durability as well as clinical impacts on the larger population beyond those included in the RCTs. Already, MABs are being studied in earlier phases of disease and will begin to answer further scientific questions around timing for starting these or other treatments. Different modes of delivery (e.g., injections instead of infusions), if possible, should ease the burden of administration. But, even with current scientific and logistic problems solved, inevitably, new questions will follow.

Looking to most other chronic and complex diseases, it is anticipated that tailored therapies will be increasingly biomarker based, tracked longitudinally, informed by genetics, and initiated at the earliest indications of disease (ideally before symptoms arise). At each innovative step on the path to better treatment outcomes, the same questions and challenges will emerge: Who will derive the greatest benefit relative to risks and why? Patients are counting on clinicians and researchers to know the answers to increasingly nuanced questions regarding treatment for the complex, heterogeneous set of diseases causing AD and related dementias. Should a fundamentally new era of dementia care emerge—an aspirational period involving early disease detection, arrest, and a functional cure—current clinicians treating persons with dementia may be the first to answer a new question: Without treatment, what would have happened to me?

## Synthesis

Reviewing Editor: Christie Fowler, University of California Irvine Decisions are customarily a result of the Reviewing Editor and the peer reviewers coming together and discussing their recommendations until a consensus is reached. When revisions are invited, a fact-based synthesis statement explaining their decision and outlining what is needed to prepare a revision will be listed below. The following reviewer(s) agreed to reveal their identity: Gernot Riedel, Abass Alavi.

The authors have described the rapidly evolving role of anti-amyloid immunotherapy and the issues that need to be addressed before widespread use of this treatment can be adopted for managing patients with AD. The review complements the ongoing debate about anti-amyloid immunotherapy making it timely and able to enhance future efforts to treat patients with Alzheimer's Disease (AD). Opinions on the questions surrounding monoclonal antibody infusion therapies are diverse, and the authors have done their best to make this submission a balanced commentary.

The authors discussion surrounding the MCID is thoughtful. The warning against using group level differences to calculate MCID and calling attention to the expectations regarding the length of time needed to reach MCID make a valuable contribution to the literature. Given the latter point, some acknowledgment should be offered that a small effect size might require an even longer trial period than the allotted 18 months. Would patient-level data have been useful in deepening this discussion?

The issue of safety is discussed by the authors, but the recent publication by Solopova et al (Nature Communications | (2023)14:8220) would make a useful addition to the references.

There should be an effort made to relate the findings in the two trials to what would be expected in normal aging. First, this would be an additional opportunity to comment on the extent to which the trial populations are representative of the population as a whole - a topic that is covered in the text. In addition, however, the placebo and antibody arms both began with individuals in early stages of the AD process. While the benefit of treatment may summate to 4-6 month delay compared to the AD-induced decline (i.e., placebo), there is still a substantial decline one might expect due to 'healthy' aging. Stated differently, do we know to what extent the therapy slows aging and to what extent it slows Alzheimer's? Some discussion of the relative rate for each would be appropriate.

The graphs/diagrams in Figure 1 are a bit misleading as they show the treatment and placebo arms continuing to diverge over time. This is based on the assumption of a constant benefit of treatment over time. This is certainly the hoped-for effect, but the data we have so far are not sufficient to prove that our hopes will be realized. The authors do acknowledge that this is the case, but they should be more forthcoming about this assumption in their discussion of the use of the STABLE equation and offer alternative perspectives where, as with cholinesterase inhibitors, the lines of cognition vs. time become parallel.

There are concerns about how accurately amyloid imaging informs us about the actual amyloid deposits in the brain (or their removal). The IDEAS project that was sponsored by CMS/Medicare concluded, for example, that amyloid imaging is suboptimal for the diagnosis and management of AD patients. It was noted that while amyloid plaques are primarily in the parietotemporal lobes, amyloid images show uptake primarily in the frontal lobes even in patients with mild cognitive impairment when there is no or minimal presence for amyloid plaques in this location. Indeed, Sevigny J et al. (Nature 537(7618):50-6.) argue that reduction of amyloid agent uptake is only noted in white matter where no amyloid plaques exist. The issue is of considerable importance as the FDA, in granting approval, relied heavily on PET imaging as a biomarker. Some discussion of this point would seem relevant to the article.
